# Cross Lingual Sentiment Analysis: A Clustering-Based Bee Colony Instance Selection and Target-Based Feature Weighting Approach

**DOI:** 10.3390/s20185276

**Published:** 2020-09-15

**Authors:** Mohammed Abbas Mohammed Almansor, Chongfu Zhang, Wasiq Khan, Abir Hussain, Naji Alhusaini

**Affiliations:** 1 School of Information and Communication Engineering, Zhongshan Institute, University of Electronic Science and Technology of China, Chengdu 611731, China; 201614010105@std.uestc.edu.cn; 2 School of Electronic Information, University of Electronic Science and Technology of China, Zhongshan Institute, Zhongshan 528402, China; 3 Department of Computer Science, Liverpool John Moores University, Liverpool L33AF, UK; w.khan@ljmu.ac.uk (W.K.); A.Hussain@ljmu.ac.uk (A.H.); 4 Department of Computer Science, School of Computer Science and Technology, University of Science and Technology of China (USTC), Hefei 230026, China; husaini@ustc.edu.cn

**Keywords:** cross-lingual sentiment analysis, multi-graph semi-supervised learning, sample selection

## Abstract

The lack of sentiment resources in poor resource languages poses challenges for the sentiment analysis in which machine learning is involved. Cross-lingual and semi-supervised learning approaches have been deployed to represent the most common ways that can overcome this issue. However, performance of the existing methods degrades due to the poor quality of translated resources, data sparseness and more specifically, language divergence. An integrated learning model that uses a semi-supervised and an ensembled model while utilizing the available sentiment resources to tackle language divergence related issues is proposed. Additionally, to reduce the impact of translation errors and handle instance selection problem, we propose a clustering-based bee-colony-sample selection method for the optimal selection of most distinguishing features representing the target data. To evaluate the proposed model, various experiments are conducted employing an English-Arabic cross-lingual data set. Simulations results demonstrate that the proposed model outperforms the baseline approaches in terms of classification performances. Furthermore, the statistical outcomes indicate the advantages of the proposed training data sampling and target-based feature selection to reduce the negative effect of translation errors. These results highlight the fact that the proposed approach achieves a performance that is close to in-language supervised models.

## 1. Introduction

With the development of Web 3.0 era, artificial intelligence (AI), increasing amount of multi-lingual user-generated content are available that expresses the users’ views, feedback or comments concerning various aspects such as products quality, services, and government policies. User-generated content contains rich opinions about several topics including brands, products political figures, celebrities, and movies. Due to the business values of the huge bulk of user-generated subsistence, sentiment analysis has received much attention in recent years.

Due to the multilingual nature of the user-generated contents, the necessity for an effective and autonomous multilingual and cross-lingual social media analysis technique is becoming vital. The majority of the existing sentiment research has been focusing predominantly on the English language, except for a few researches exploring other languages. Various well-regarded sentiment resources, i.e., lexicons and labeled corpora, are constructed for the English language. Research progress in other global languages is limited due to the lack of such sentiment resources [1,2,3,4,5,6,7,8]. The manual development of dependable annotated sentiment resources for each poor resource language and its domain is a time-consuming and intensive task. In order to overcome the annotation cost, various solutions have been proposed in the literature to exploit the unlabeled data in target-language (this is called semi-supervised learning) [1], or to explore translated models and/or data available in other languages (this is called transfer learning) [3,4,5,9]. The lack of these annotated resources in the majority of languages motivated research toward cross-lingual approaches for sentiment analysis. Language Adaptation (LA) or Cross-Lingual Learning (CLL) is a particular example of Transfer Learning (TL), that leverages the labeled data in one or more related source languages to learn as a classifier for unseen/unlabeled data in a target domain. More specifically, the leveraging of sentiment resources from rich language to predict sentiment polarities of a poor-resource language text is referred to as Cross-Lingual Sentiment Classification (CLSC). Language with the availability of rich and reliable resources is usually referred to as ‘source language’, while a low-resource language is referred to as the ‘target language’. Despite the fact that sentiment analysis has received notable attention from the research community, there are limited works that focus on cross-lingual sentiment analysis. The difficulty of handling cross-lingual sentiment analysis comes from various sources such as loss of polarity during machine translation, cultural disparity, feature divergence, and data sparsity. In addition, the noisiness and informal nature of the social media text poses additional challenges to cross-lingual sentiment analysis.

Supervised Cross-Lingual (SCLL) as well as Semi-Supervised Learning (SSL) are commonly used approaches to control sentiment analysis in poor resource languages with little to no labeled data available [9]. SCLL techniques attempt to make use of current annotated sentiment resources from opulent language domain (i.e., genre or/and different topics). These approaches employ machine translation (from target to source languages, or from source to target, which are referred to as bidirectional), bilingual lexicons or cross-lingual representation learning techniques with parallel corpora to project the labeled data from source to targeted language [1,3,9,10]. It should be noted that state-of-the-art CLSA techniques suffer from insufficient performance due to the low machine translation quality as well as cultural and language divergence [3,4,9,11,12] (i.e., various sentiment expressions and social culture). The success of these approaches is largely dependent on how similar the projected source data and target language data are. In contrast, SSL techniques such as co-training, self-training, and active learning for cross-lingual sentiment classification, rely on a small quantity of labeled data from the same domain, which necessitates additional annotations. However, SSL techniques suffer from data sparseness as the small amount of labeled data used cannot cover all target test data topics. Additionally, SSL techniques cannot reasonably use large translated resources from rich languages. Therefore, discovering methods to exploit translated labeled data sets and unlabeled target data to enhance classifier performance has recently become a popular research topic.

In this paper, an efficient integrated supervised and semi-supervised learning is proposed in order to address the cross-lingual classification issues. The critical idea is to develop a cross-lingual learning model that can overcome the disadvantages of both SCLL and SSL via fusing sentiment knowledge-translated labeled and target unlabeled from multiple sources. The aim is to incorporate an extended supervised learning model trained over the selected translated labeled data samples along-with semi-supervised models learned from the target unlabeled data, to achieve superior performance. The paper investigates several research questions that are relevant in addressing cross-lingual sentiment analysis, including: (1) Which direction of SCLL or SSL is better with scarce resource languages? (2) Could translated sentiment sources be employed together with target data to solve cross-language analysis tasks successfully?

To summarize, this work has a number of contributions. First, it proposes two-dimensional noisy data reduction. In the horizontal dimension, a new cluster-based meta-heuristic sample selection method is proposed to select the optimal, informative and representative training sample. The aim is to avoid noisy examples in order to achieve the target classification best performance. In the vertical dimension, a novel modification of feature selection algorithms is proposed to select features from the translated source data set not only based on their association with classes but also associated with target data. This means that features or opinion expressions are excluded even if they are discriminating, and are only source language-specific features. Only features that are discriminating and related to the target language are chosen. Secondly, this work proposes a new integrated model where an ensemble model trained over the translated data is integrated with a semi-supervised model trained over the target data at the learning phase. The target test data is then passed to the trained integrated model, which is responsible for classifying test data instances that are similar to the source data and passing them to the semi-supervised model. So, the integrated model fuses knowledge from translated data, and simultaneously, uses target language data to handle the divergence of data distribution.

The remainder of this paper is organized as follows. Section 2 shows related studies on cross-lingual sentiment analysis. The proposed method is presented in Section 3. The experiments’ methodology and experimental results are presented in Section 4 and Section 5, respectively. Conclusion and further works are illustrated in Section 6.

## 2. Related Studies

Cross-lingual sentiment analysis is introduced to alleviate the problem of developing training data and building separate models for different languages and domains. The following subsections summarize the challenges and existing approaches for cross-lingual sentiment analysis.

### 2.1. Challenges of CROSS-LINGUAL Sentiment Analysis

Cross-lingual sentiment analysis task shows many challenges. Loss of polarity, cultural disparity, feature divergence, and data sparsity are all considered as challenges [1,4,9,10,12]. However, loss of polarity or the issue of labeling mismatching caused by the erroneous machine translation of source language training data severely deteriorates the analysis performance [3]. Previous work predominantly relies on machine translation engines or bilingual lexicons to project data from the source language to the target language [1,2,13]. Machine translation quality is still low and far from satisfactory [1,2,9]. Additionally, machine translation may result in the mistranslation of a word into something with a totally distinct meaning, which can negatively affect the quality of the projected source language labeled data. However, this is not a factor in performance degradation. On the other hand, cultural disparity is a major difficulty in cross-lingual sentiment analysis, despite translation quality [14]. Each language has its own particular way of expressing sentiments and writing styles. Even in the scenario of expressing a comparable idea, there can be a huge disparity in the vocabulary and metaphor within the context of various languages, leading to a far smaller word and phrase intersection between native expressions and translations [9]. Furthermore, feature distribution, which refers to instances of mismatching between the knowledge examples that the classifier acquired in training phase, contains many source and target language-specific features which need to be categorized. From a sentiment analysis perspective, this relates to the cross-domain sentiment analysis situation where training and test data are drawn from various domains, therefore making it a hurdle for traditional semi-supervised and supervised classification algorithms to attain satisfactory sentiment classification [10,14]. Additionally, using machine translation, auto-translated data contains a vast number of irregular words and meaning-less terms as a result of translation errors. References [15,16] indicated that incorrect translations show an increase of noisy features and sparseness. This implies that poor translation quality results in generating noisy features that ultimately provides poor classification accuracy.

### 2.2. Main Techniques for CROSS-LINGUAL Sentiment Analysis (CLSA)

Despite the fact that sentiment analysis has received a significant amount of attention, very few methods have been put forward for cross-lingual sentiment classification in various methods. First, translation-based methods are dependent on machine translation or bilingual dictionaries [17,18] to project the annotations from the source language into the target language, or vice versa. A classification model is then trained over the target data with projected annotations [2,13]. Earlier works utilize the bilingual dictionaries to transfer the sentiment features from one language to another. For example, in [17] a lemmatized form of English terms from ‘Opinion Finder’ was extracted and translated with two bilingual dictionaries to create a subjective analysis lexicon. Authors in [18] used a bilingual dictionary to create a bilingual word pair that was extracted from high-occurrence terms contained within the source and target language. The word pair is then utilized to extend the Structural Correspondence Learning Approach (SCL) proposed by Blitzer et al. [19] from cross-domain to cross-lingual adaption. The primary disadvantage of bilingual dictionaries is that they are unable to capture a word context during translation, as each word may possess a distinct meaning in a variety of contexts. Thus, it is likely to generate a negative effect on the accuracy of sentiment analysis in the target language [1,20]. The recent innovations in machine translation have motivated researchers in sentiment analysis to utilize freely available MT services such as Google translator and Bing translator. For example, the researchers in [2] showed that machine translation is a reliable tool to generate sentiment analysis resources for multilingual sentiment analysis. More recently, [13] investigated whether machine translation could be employed to generate reliable training data for emotion analysis. For this purpose, they employed Google engine to translate the original text from the source language. In [21], a direct transfer model, namely, adversarial deep averaging network, is trained to transfer the knowledge learned from source labeled data, whereby the model attempts to create language invariant bilingual representations. Despite recent advancements in machine translation tools, researchers are still unwilling to take advantage of such technologies as they have concerns about the capabilities of machine translation systems to preserve the sentiment information across languages.

The second method can be referred to as domain adaptation methods that attempt to deal with the cross-lingual using cross-domain adaptation. For instance, Structural Correspondence Learning (SCL) [19] was employed for CLSA in several studies, including [18,22]. Such methods provide correspondence among words taken from two different languages utilizing ‘pivot features’ which represents the set of frequent features provided in both languages. In this case, a pair of words is utilized from source and target languages as pivot features to model the correlation among these features and other words using leaner classifiers and unlabeled data set which can be used as an independent language predictor. Additionally, Li et al. [22] employed the distributed representation of words to build one-to-many mappings between the pivot features in the source language (English) and those in the target language (Chinese). The direction aims to deal with feature divergence or domain mismatch between translated resources and target data. Unfortunately, accuracy degradation naturally occurs even in cases of perfect MT, because of the cultural disparity, i.e., language divergence. Also, there is no regular method for pivot selection and therefore, these methods cannot guarantee performance in all situations.

The third direction of CLSA is cross-lingual representation learning methods. These methods prompt language-independent features to bridge the cross-lingual distinction in the original word-level representation space and build connections across various languages [23]. The majority of these methods employ parallel corpora to train bilingual word embedding to obtain aligned inputs for learning feature extraction that works with both languages [24]. Recently, Chen et al. [25] proposed the idea of joint two-view convolutional neural networks (CNNs) to grasp the connection from opinionated documents across languages. Their method begins by projecting the extracted parallel sentiments connection into a bilingual sentiment relation space. Then, it captures the connection by subtracting them with an error tolerance. In [26], sentence-aligned corpora were used between a pair of resource-rich and resource-poor languages. The assumption is that the system is reliant upon the semantic similarity between different phrases, which implies sentiment similarity in most sentences. Later, [27] presented a model that could be used to learn distributed representations through a hierarchy of network architectures.

Recently, [28] demonstrated that word embedding can be enriched for sentiment information without the necessity of a labeled corpus. This enrichment improves the performance across sentiment and non-sentiment-related tasks. The primary disadvantage of the cross-lingual representation method for CLSA is the need for large parallel corpora, which is difficult to obtain, especially when high quality is required.

Semi-supervised learning direction is followed by researchers when needing to deal with cultural disparity challenges in which each language has its own sentiment expressions. Researchers used unlabeled data in the learning process to strengthen classification performance [29,30]. A bilingual co-training approach is exploited to leverage annotated English resources for sentiment classification in Chinese. A machine translation service was utilized to translate labeled English documents (training documents) and unlabeled Chinese documents (testing documents) into each other. The author used two different views (English and Chinese) and employed the co-training approach for a classification problem. Later, unlabeled data for the target language are incorporated into the learning process via a bi-view framework proposed by [20]. The key idea is to enrich training data through the addition of the most confident automatically labeled examples, as well as a few of the most informative manually labeled examples from unlabeled data in an iterative process.

Finally, several works have recently been introduced to incorporate sentiment information. Bilingual Sentiment Embedding (BLSE) [31] is proposed to jointly optimize and represent both semantic information and sentiment information given comparably little bilingual lexicon and an annotated sentiment corpus.

Related to this work, some researchers have tried and are trying to solve the issue of different term distribution between the training (translated data from source language) and test data in CLSC-incorporating unlabeled data from the target language using a semi-supervised learning approach. A number of research studies use the supervised or semi-supervised classification trained on translated data to classify the unlabeled target data. The most solid instances are used as seeds for a semi-supervised model. Due to the significant vocabulary gaps between source data and target data, the supervised model incorrectly classifies instances of the seeds that, in turn, were used in the semi-supervised model. When the initial classifiers are not sufficient, this can increase the examples that are incorrectly labeled within the training set. Hence, using noisy examples will decrease the accuracy of the learning model as well as the performance of each classifier. It should also be noted that confident classified examples may not essentially provide information during the learning process. Hence, the addition of these examples may not be useful from the classifier point of view.

Both [1,32] used active learning with semi-supervised learning to select the most confident and most informative training samples to be added. The main disadvantage of this method is that it requires human intervention from experts in the target language, and this can be regarded as the semi-automatic annotation of the data set. Considering these problems, several factors are dwelt on within the proposed research study. First, the model proposed utilizes the available sentiment resources, labeled translated data set, labeled seed from the target language, and unlabeled data from the target language. Second, the proposed model integrates a supervised model with semi-supervised at the learning phase. Third, this work brings forward a new clustering-based bee-colony sample-selection method to choose the optimal, informative, and noiseless representative training sample to achieve superior performance of the target classification. This work proposes target-based feature-selection algorithms to select features from the translated sources data set based on their association with classes and with the association with target data. This means that feature or opinion expressions that are discriminating and associated with the source language specific-features, are excluded. Hence, only features that are discriminating and related to the target language are selected.

### 2.3. Main Techniques for Instance Selection and Feature Weighting

The handiness of vast amount of source target training data has necessitated the development of accurate and fast algorithm capable of discovering underlining information within the big data. Most of the proposed algorithm focus on randomly selected subset of data. Recent approaches for training sample selection are classified into instance-based methods and feature-based methods. Instance-based methods focus on weighting individual instances based on their importance to the target domain [33]. Feature-based methods are based on projecting, mapping, as well as representing features in a way that the source classifier can achieve well using the target data. A feature-based method presented in [34] uses a greedy algorithm for the selection of a subset of features from a source data matrix which approximates the features of a target matrix. Xia [5] identifies the most relevant target domain samples and uses them as the training dataset. In [35,36], a semi-supervised learning technique called PU learning is used to identify and weight these instances. The performance is evaluated on cross-domain sentiment classification. Different samples in the source training are provided with different weights during the base model training to make the distribution of training dataset resemble the distribution of target data. In Xia et al. [36], the authors extend this work by taking both sample selection bias and sample selection variance into consideration for the domain adaptation. To improve PU-learning, ref [37] proposed an approach called PUF that additionally selects reliable negative instances through the fuzziness of the instances.

## 3. The Proposed Method

As discussed earlier, the primary aim of the proposed method is to leverage the available sentiment resources, translated resources, and target language resources to strengthen the sentiment analysis performance and tackle the language gaps. The key idea presented in this work by integrating SL with SSL is that the supervised model is responsible for classifying target data that are similar to training data, i.e., translated data. Those target instances that are classified with low confidence by the supervised model are passed with prior information to a semi-supervised model trained over the target data for classification. The concept of transferring previous information is to combine the influence of translated resources with the influence of target data and to reduce the time complexity of graph-based algorithms by accelerating them to convergence.

To benefit from translated resources effectively, two levels of filter are proposed to minimize the translation noise. The first level (horizontal level or sample selection) aims to select optimal, informative and representative training samples and avoid noisy examples to achieve the best target classification performance. The second level (vertical level or feature selection) uses a target-based feature selection algorithm to select features that are discriminative and simultaneously associated with the target data. Generally, the proposed method consists of (1) clustering-based bee-colony training sample selection, (2) target-based feature selection, (3) ensemble supervised learning, (4) integrating prior supervised information with semi-supervised learning, (5) multi-graph semi-supervised learning. The details of each component are shown as follows.

### 3.1. Clustering Based on BEE-COLONY Training Instance Selection

Instance selection (sample selection) is one of the important components of cross-lingual sentiment classification because a rich-resource language such as English has many datasets from different domains, each containing large size of labeled reviews. Therefore, it is quite easy to obtain a large collection of labeled reviews along-with machine-based translation. However, only some of them may be useful for training a desired target-language sentiment classifier. Therefore, it is important to identify the samples that are the most relevant to the target domain. Under this circumstance, instance selection is necessary for training an effective classifier [35,36,38]. Unlike domain adaptation, instance selection within the cross lingual adaptation has an additional aim to filter out the noisy instances from a selected dataset. Such noisy instances (or outliers) within the source translated dataset are usually generated due to the language gap and translation errors.

Existing multi-source or single source cross-language methods frequently utilize the entire translated source data and ignore the selection of appropriate data instances from the translated source data to be used for adaptation. Nonetheless, assuming the availability of a single-source language or multi-source languages, it is critical to adapt and choose the most efficient training instances that are suitable for the target language. This is a critical issue that has received little attention. Because of the vocabulary gaps between translated data and target data, the supervised classifier will not accurately classify the target data. To overcome this problem, this section describes an instance selection algorithm to select the high-quality training data from the translated source language data that is used to train the supervised classification model. The main objective of this component is to select the optimal training samples to achieve efficient performance of the target classification. In this phase, the top ranked source domain clusters are selected as a source training set. Given that the translated data instances from source(s) languages and domains, a new cluster-based bee-colony meta-heuristic instance selection algorithm is proposed to discover the best training sample from the source language.

### 3.2. Clustering Target Language Data

The algorithm divides the target language into Q number of clusters, each represented as Cq, q = {1, 2, …, Q}. The aim is to utilize these clusters to select representative source training data. To overcome the limitations of k-based clustering where the number of clusters must be predefined, this work introduces radius-based clustering. The step-by-step flow of the proposed algorithm is summarized below:

(i) Inferring a target data similarity matrix: Given an unlabeled target data set consisting of the feature vectors of m unlabeled reviews, $U=\left\{u_{1},\ldots ,u_{m}\right\}$, The similarity matrix element $S_{\mathrm{ij}}$ is computed between each pair of the unlabeled reviews ($u_{i},\;u_{j}$) from the target language dataset using cosine similarity measure as in Equation (1):
  (1) $$S_{\mathrm{ij}}=\cos\left(u_{i},\;u_{j}\right)=\frac{\left|u_{i}\right|*\left|u_{j}\right|}{\sqrt{u_{i}^{2}{\;*\;u}_{j}^{2}}}$$
  The constructed similarity matrix is built through computing pair-wise similarity between the target set instances:
  (2) $$\left[\begin{array}{ccc} S_{11} & \ldots & S_{1m}\\ \vdots & \ddots & \vdots \\ S_{m1} & \ldots & S_{\mathrm{mm}} \end{array}\right]$$
(ii) Estimating reviews density: A random number r is selected where 0.5<r≤1. The algorithm then calculates the density of each unlabeled review $u_{i}$ of the data as:
  (3) $$\mathrm{Density}\left(\mathrm{ui}\right)=|\left\{u_{j}:S_{\mathrm{ij}}\geq r\;;\forall j\;\right\}$$
  where $S_{\mathrm{ij}}$ is the cosine similarity $\cos\left(u_{i}\;,u_{j}\right)$ between two feature vectors of review $u_{i}$ and $u_{j}$.$\;\mathrm{Density}\left(u_{i}\right)$ is the density of review, $u_{I}$ is the set of all reviews whose cosine similarity such that the review $u_{i}$ is greater than r. After computing the density function for all reviews, a review which has the highest density (i.e., $\mathrm{Density}\left(u_{i}\right))>\mathrm{Density}\left(u_{j}\;\right);\forall j$) is then chosen as the seed of the first $S_{j1}$, i.e., review which has most similar reviews, and all reviews in $S_{j1}$ density set $\left(\left\{d_{j}:\mathrm{dist}\left(u_{i}\;,u_{j}\right)\leq r\;;\forall j\right\}\right)$ are removed from the data set.
(iii) The centroid of each cluster: Given the selected review and all reviews in its density set, the centroid of a formed cluster is computed as average of the feature vectors of all cluster members (i.e., density set members), as shown in the equation below:
  (4) $$\mu_{f}=\frac{1}{\left|C_{f}\right|}\overset{\left|C_{f}\right|}{\underset{\propto =1,}{\sum }}u_{\propto }$$
  where $\mu_{f}$ is the centroid of the formed cluster,$\;\left|C_{f}\right|$ is the number of reviews in the cluster f and $u_{\propto }$ is the feature vector of review $u_{\propto }.$
(iv) Selection Q optimal target clusters: Repeat step *(iii)* and step *(iv)* to continue selecting the subsequent clusters as long as the algorithm continues to find documents in the data set.

### 3.3. Improved Artificial BEE-COLONY Training Selection

Algorithm 1. In this step, the goal is to craft an approach for selecting a training sample from the source language. This means finding an optimal translated source data to be utilized as training data for the target language. The selected sample is aimed to be representative, with fewer translation errors, and suitable for the target domain. Also, sample instance topics must have the same topic distribution as the target language data, i.e., contains data that cover more topics in the target data in the concept space.

To start with, the artificial bee colony (ABC) produces a randomly distributed which will be the population of SN solutions (i.e., positions of the source food) using search space, in this case the SN represents the size of engaged bees. Each solution $x_{k}$ is a D-dimensional vector in which k represents the number of the solution with k=1,2,…,SN. Here, D is the number of translated reviews from a single source. All solutions generated in this phase are collected using (5):

(5) $$x_{\mathrm{kz}}=\theta_{k,z}$$

$\theta_{k,z}$ is a random number between [0, 1] while z is the number of a translated reviews from the given source z=1,2,…,D. After the initialization phase, each employed bee’s position is discretized to reveal the selected and omitted reviews. Specifically, employed bees are represented as a vector of {0,1} defining whether a review $d_{z}$ in the translated dataset from a particular domain, is selected or not, as shown in (6)

(6) $${\;x}_{k,z}=\{\begin{array}{cc} 1 & {\;\mathrm{if}\;d}_{z}\;\mathrm{is}\;\mathrm{selected}\;\mathrm{in}\;\mathrm{solution}\;k\\ 0 & \mathrm{otherwise} \end{array}$$

This means changing the real position to a discrete one; each $x_{k,z}$ is set to a binary number 0 or 1. The following equations are applied to map each $x_{\mathrm{ij}}$ to be zero or one:

(7) $$x_{k,z}=S\left(\theta_{k,z}\right)=\frac{1}{1+e^{-\theta_{k,z}}}$$

Then, the artificial bee colony calculates the amount of nectar in each food source depending on the quality of the associated solution. Given g target data clusters and the calculated centroid $\mu_{f}$ f={1,…,g} of those clusters calculated in the previous section. To calculate the fitness $F_{k}$ of each solution (employee bee) ${\;x}_{k}$, the algorithm does the following steps:

(1) For each review $d_{a}$ from the solution ${\;x}_{k}$( selected sample from the source), the algorithm finds the maximum similarity of review and each centroid $\mu_{f}$ of the g target clusters as follows:
  (8) $${\mathrm{ms}}_{a}=\underset{\forall f}{\max\;}\cos\left(\left(d_{a},\mu_{f}\right)\right)$$
  where ${\mathrm{ms}}_{a}$ can be defined as the maximum similarity of a review $d_{a}$ and the target data.
(2) Then, the fitness of the solution ${\;x}_{k}$ is defined as average of total maximum similarity of all of its reviews and calculated as follows:
  (9) $$F_{k}=\frac{\sum_{d_{a}\in {\;x}_{k}}{\mathrm{ms}}_{a}}{\left|{\;x}_{k}\right|}$$

$\left|{\;x}_{k}\right|$ is the number of selected reviews in the solution $x_{k}$. An onlooker bee assesses the information of the nectar for all employed bees and selects the source of the food based on the probability of the nectar quantity. This probability value is determined based on the following formula:

(10) $$P_{i}=\frac{F_{i}}{\sum_{i=1}^{\mathrm{SN}}F_{i}}$$

To provide diversity for the population, the onlooker is required to find local search with improved nectar resources around the corresponding resources for each generation. Global artificial bee colony introduces the global optima into the search formula of artificial bee colony for improving the exploitation based on the following formula:

(11) $$\bar{x_{k,z}}=x_{k,z}+\theta_{k,z}\;*\;\left(x_{k,z}-x_{h,z}\right)+\beta \;*\;\left(x_{z}^{\mathrm{glob}}-x_{k,z}\;\right)$$

where $\bar{x_{k,z}}$ is a new value of review $d_{z}$ in the generated solution $x_{k}$, $x_{h,z}$ is the value of review $d_{z}$ in the solution $x_{h}$ which h is a random number between 1 and D and not equal to k and $x_{z}^{\mathrm{glob}}$ is the value the value of review $d_{z}$ in the best global solution $x^{\mathrm{glob}}$. Onlooker bees as well as employed bees complete manipulation for the search area, and consumed food sources are replaced with a new one using the artificial bee colony algorithm with the scout bees during the discovery process. If the position is not enhanced as a previously determined cycle number, the food source is acknowledged as abandoned. In this case, a previously concluded cycle number is considered the “limit” for abandonment. With this scenario, three control parameters in ABC are utilized: the number of food sources (SN), equals to the number of employed and onlooker bees, the limit value and the maximum cycle number (MNC). If an abandoned solution is assumed to be $x_{k}$ and (z = 1, 2 … D), the scout goes to search for a new replacement solution, as in Equation (12).

(12) $$x_{k,z}=x_{k,z}^{min}+rand\left(0,1\right)*\left(x_{k,z}^{max}-x_{k,z}^{min}\right)$$

where $x_{k,z}$ is the value of review $d_{z}$ in the solution $x_{k}$, $x_{k,z}^{\min}$ and $x_{k,z}^{\max}$ are the lower and upper bounds of the value of review $d_{z}$ in the generated solution $x_{k}$, respectively.

The performance of new food source is compared with the previous one. When the new food source has an equivalent or more amount of nectar than the previous one, new one will substitute the old food source in memory. Otherwise, the old one holds its memory position. This implies that a greedy selection mechanism is used to make selections among the old source and that of the candidates.

**Algorithm 1.** ABC algorithm Pseudo code**Input:** Translated Training Data, Q optimal target clusters, S centroids of target clusters **Output:** Optimal Training DataFor each Cluster C_i_ in Q target clusters  (1) Generate the initial population { $x_{1},\ldots ,x_{n}\}$
 (2) Assess the fitness of the population using Equation (9) (3) Let cycle to be 1 (4) Repeat (5) FOR each solution (employed bee)    Begin    Find new solution from $x_{i}$ with Equation (11)    Determine the fitness value using Equation (9)    Employ greedy process    EndFor (6) Find probability values $P_{i}$ for the solutions utilizing Equation (10) (7) FOR every onlooker bee     Begin    Choose solution based on $P_{i}$    Generate new solution from $x_{i}$ utilizing Equation (11)    Determine the fitness value using Equation (9)    Employ greedy selection process    EndFor  (8) If abandoned solution for the scout is determined,  Begin  swap it with a new solution   randomly generated using Equation (12)  EndIF (9) Remember the best solution up to this point (10) increase cycle by 1 (11) Until cycle equals to MCN

### 3.4. Target-Based Feature Selection Methods

Algorithm 2. In the previous step, a sample selection or horizontal noise removal is used, which selects a sample of best training instances that are appropriate for the target language. This has been performed at instance level. In the following step, these reviews are passed through machine translation using Google translation, preprocessing and feature selection components. In traditional feature selection methods, features are selected based on their class weights. However, not all features included in these instances are useful for target language sentiment analysis. For instance, a word that cannot be translated to the target language by the machine translation appears in its original language in the translated text. These words should be removed even if they are selected by the feature selection method. To design a target-based feature selection, we introduce target-feature weighting methods for selecting features that are discriminating and suitable for the target language. This is called ‘vertical noise removal’. Features are chosen according to two factors (a) to their class weights and (b) target-language weights. Firstly, this work evaluates a pointwise mutual information feature weighting method for measuring its correlation with source data classes. The pointwise mutual information feature selection method selects features for each class according to the co-occurrence measure between a feature $f_{j}$ and a class $c_{i}$. The pointwise mutual information (nPMI) between the feature and its classes is calculated using (13).

(13) $$\mathrm{nPMI}\left(\mathrm{class}=c_{i},f_{j}\right)=\frac{\mathrm{PMI}\left(c_{i},f_{j}\right)}{\sum_{f_{k}}\mathrm{PMI}\;\left(c_{i},f_{k}\right)}$$

After that, features are weighted in target data based on their occurrence in the target data using (14):

(14) $$\mathrm{Tw}\left(f_{j}\right)=\frac{f\left(T,f_{j}\right)}{f\left(T,f_{j}\right)+f\;\left(S,f_{j}\right)}$$

where $f\left(T,f_{j}\right)$ and $f\left(S,f_{j}\right)$ are the term frequency-inverse document frequency (TFIDF) of feature $f_{j}$ in both sources translated data and target data.

**Algorithm 2.** Algorithm for integrating prior supervised information with semi-supervised training. **Input:***UT* Test Unlabeled data from the target language,
    *LS*: Selected labeled training sample from Source language.

**Output:** Unconfident Group *UG*, Prior Label matrix *PL*, confident group *CG*
Begin
(1)
Train classifier C1 on LS. (2)
Train classifier C2 on LS. (3)
Train classifier C3 on LS. // *C1, C2 and C3* used to predict class label and calculate // the prediction confidence of each example in U (4) For Each (Example u_i_ in UT)
 Begin
  $P_{1}\leftarrow {\mathrm{Predict}\text{\_}\mathrm{label}(c}_{1}{,u}_{i})$
  $P_{2}\leftarrow {\mathrm{Predict}\text{\_}\mathrm{label}(c}_{2}{,u}_{i})$
  $P_{3}\leftarrow {\mathrm{Predict}\text{\_}\mathrm{label}(c}_{3}{,u}_{i})$
 // calculate average confidence values
 $\;\mathrm{ACV}\leftarrow \mathrm{ensemble}\left(P_{1},P_{2},P_{3}\right)\;$
  IF ( ACV >γ)
 Begin
  CG←CG∪(u,l)
 Else
  UG←UG∪u
   PL←ACV 
 ENDIF
 ENDFOR (5)
 Call Semi−Supervised (UG, PL)
End RETURN CG, UG, PL

### 3.5. Ensemble Supervised Learning

The final prediction is performed using an ensemble approach by integrating the outcomes from a supervised and a semi-supervised model. The supervised model is trained using a selected sample from the translated source data while the semi-supervised graph-based model learned the patterns within the target data. The main objective is to strengthen the classification performance and reduced complexity of the graph-based model.

In the ensemble model, classification is performed using the weighted voting to combine the predictions from multiple algorithms as in Equation (15):

(15) $$H\left(x\right)=\overset{T}{\underset{i=1}{\sum }}\alpha_{i}h\left(x_{i}\right)$$

where h is a classifier, H is …., alpha is the Naïve Bayes, maximum entropy, and logistic regression are utilized as base classifiers. Each weak classifier offers an output prediction, h(xi), for every target test sample. Every base learner has a weight, αi so that the error sum is minimized.

Naïve Bayes uses the Bayes’ theorem with strong or naïve independence assumptions for classification. Provided with feature vector table, the algorithm calculates the posterior probability that the document belongs to distinct classes and assigns the document to the class that has the highest posterior probability. To classify the most probable class c* for a new document d, NB computes Equation (16):

(16) $$C^{*}=\arg\underset{c}{\max}\;p(c|d)$$

The NB classifier calculates the posterior probability as in Equation (17):

(17) $$p\left(c_{j}{|d}_{i}\right)=\;\frac{p\left(c_{j}\right)p\left(c_{j}{|d}_{i}\right)}{p\left(d_{i}\right)}$$

A detailed explanation of the NB classifier can be found elsewhere. Maximum Entropy (ME) classifier estimates the conditional distribution of the class label $c_{i}$ given a document $x_{j}$ using the form of an exponential function with one weight for each individual constraint as in Equation (18):

(18) $$P_{\omega }(c_{i}|\;x)=\frac{1}{z\left(x\right)}e^{\left\{\omega_{i}\;f\left(c_{i}\;,\;x\right)\right\}}$$

(19) $$f\left(c_{i}\;,\;x\right)=\{\begin{array}{cc} 1 & \;\mathrm{if}\;c=c_{i}{\;\mathrm{and}\;x\;\mathrm{contain}\;w}_{k}\\ 0 & \;\mathrm{otherwise}\; \end{array}$$

where each $f_{i}\left(c_{i}\;,\;x\right)$ represents a feature, $\omega_{i}$ is the weight to be determined through optimization, and Z(x) is a normalization factor. $P_{\omega }(c_{i}|\;x)$ is estimated for each class, and the class with the highest probability value will be selected as the class of document x. $f\left(c_{i}\;,\;x\right)$ as an indicator function returns one only when the class of a particular document is $c_{i}$, and the document contains the word $w_{k}$. Further details about ME can be found in [37]. Logistic regression defines the predicted probability as in Equation (20):

(20) $$f\left(x\right)=P\;(c_{i}|\;x)=\frac{e^{\beta_{0}+\beta_{1}f_{1}+\ldots +\beta_{k}f_{k}\;}}{1+e^{\beta_{0}+\beta_{1}f_{1}+\ldots +\beta_{k}f_{k}\;}}$$

where the coefficient $\beta_{i}$ controls the effect of the feature. The further a $\beta_{i}$ drops from 0, the more dramatic the effect of the feature $f_{i}$.

The diversity of the ensemble classifier is generated by several factors:

(1) Using different types of base classifiers.
(2) Selecting samples that contain instances generated randomly, and
(3) Selecting samples that are distributed in a representative and informative way. The final prediction output of the ensemble model is obtained by averaging the confidence values for each label.

### 3.6. Integrating Prior Supervised Information with Semi-Supervised

To leverage the benefits of the source language annotated resources through supervised approaches and the unlabeled examples from the target language through semi-supervised learning, we use an integrated model that combines both approaches. The output from the ensemble model (described in the previous section) is clustered into two categories on the basis of the obtained average confidence values. The average confidence of an example is calculated by averaging the confidence of the majority classifiers in predicting the label of that example. The first type of output is a group of all test instances that have been assigned to their classes based on high average confidence values, i.e., a group of the most confident, positive examples and the most confident negative examples. Classes associated with these test instances are considered as their final class predictions.

The second group (i.e., unconfident group) represents the set of test instances that have been assigned low average confidence values because they contain target language opinion expressions. In other words, they have different term distribution with the translated training data set. Instances of the unconfident group are transferred along with their associated values to the semi-supervised learning module (next section).

### 3.7. SEMI-SUPERVISED Learning

**Figure 1:**
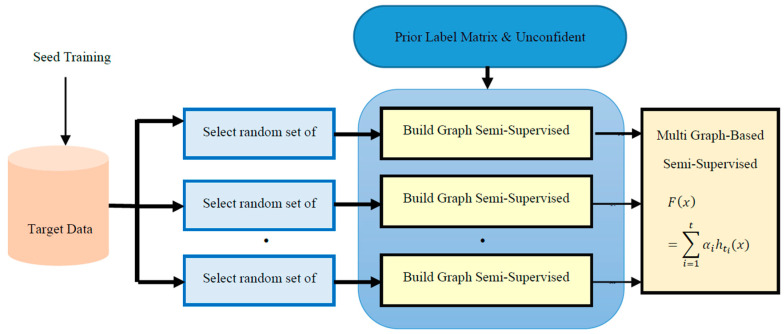
Multi-Graph Semi-Supervised Learning with Prior Label Information.

As mentioned in the previous section, the semi-supervised model is responsible for classifying test instances that have been categorized with low average confidence in ensemble model. The idea is that they contain target language opinion expressions i.e., they have different term distribution with the translated training data set. Given a data set $=X_{l}+X_{u}\;\in {\;R}^{d}$, where, d is the dimension of the feature space. $X_{l}=\left\{x_{1},\ldots ,x_{n}\;\right\}$ is a labeled seed set from the target data. $Y^{\left(l\right)}$ is the $R^{n*2}$ label matrix of these seed sets. For each review i from the seed set, $Y^{\left(l\right)}\left(i,0\right)$ is 1 if $x_{i}$ is labeled as negative, and $Y^{\left(l\right)}\left(i,1\right)$ is 1 if $x_{i}$ is labeled as positive. $X_{u}\;=\left\{x_{n+1},\ldots ,x_{n+m}\right\}$ is an unlabeled unconfident set with prior probabilities from the supervised model. $Y^{\left(\mathrm{up}\right)}$ is the label matrix for the test data set. $Y^{\left(\mathrm{up}\right)}$ is $R^{m*2}$. N=n+m is the size of the total data set. For traditional graph-based method, both $Y^{\left(\mathrm{up}\right)}\left(i,0\right)$, and $Y^{\left(p\right)}\left(i,1\right)$ are initialized to 0 for each review from the test set. In our integration algorithm, $Y^{\left(\mathrm{up}\right)}\left(i,0\right)$, and $Y^{\left(\mathrm{up}\right)}\left(i,1\right)$ are the prior probabilities for positive and negative classes passed from the supervised model. The multi-graphs algorithm as shown in Figure 1 is described below:

Step (1) Each review is represented as a feature vector.
Step (2) Initialize the label matrix $Y=\begin{array}{ccc} Y^{\left(l\right)} & ; & Y^{\left(\mathrm{up}\right)} \end{array}\in R^{N*2}$ for the labeled data set. $Y^{\left(l\right)}$ and $Y^{\left(\mathrm{up}\right)}$ is described above.
Step (3) Randomly select f features from all features
Step (4) Graph construction:
  (a) Each $x_{i}$ a labeled or unlabelled review, a node is assigned. Allow $V=\left\{v_{1},\ldots .,v_{n}\right\}$ to be a set of vertices.
  (b) K-NN node calculation: To construct the graphs, the nearest neighbor method is employed. Two nearest k neighbors set a review of $x_{i}$ and is determined where ${\mathrm{Knn}}_{u}\left(x_{i}\right)$ is a set of K nearest unlabeled neighbors, and ${\mathrm{Knn}}_{l}\left(x_{i}\right)$ is a set of K nearest labeled neighbors of $x_{i}$. A review $x_{j}$ is assigned to one of the k nearest neighbors set of review $x_{i}$ if their edge weight $w_{\mathrm{ij}}$ between their feature vectors is greater than ε. The weight of an edge $w_{\mathrm{ij}}$ is defined with the Gaussian kernel:
    (21) $$w_{\mathrm{ij}}=\exp\left(-\frac{{\mathrm{xv}}_{i}-{\mathrm{xv}}_{j}{}^{2}}{\sigma^{2}}\right)$$
    where ${\mathrm{xv}}_{i}$ is the feature vector of review x_i_, Weight matrix $W=\left[\begin{array}{cc} W_{\mathrm{LL}} & W_{\mathrm{LP}}\\ W_{\mathrm{PL}} & W_{\mathrm{PP}} \end{array}\right]$ is constructed.

Step (1) Run semi-supervised inference on this graph utilizing label propagation:
  (22) $$Y_{p}\;\leftarrow \;\left(1-\;\gamma \right)W_{\mathrm{pp}}\hat{{\;Y}_{p}}+{\;\gamma \;W}_{\mathrm{PL}}\;\hat{Y_{L}}.$$
  Finally, Normalize $Y_{p}$
  Repeat the above steps n times from step 3 to build n trained semi-supervised models
  Each trained with different feature set;
Step (2) The n Semi-Supervised classifier vote to determine the final labels for the unlabeled data $Y_{p}$.

## 4. Experimental Design

This work is evaluated using a standard evaluation data set for cross-lingual sentiment classification from English to Arabic presented in [3]. The Amazon corpus [19] is used as a benchmark dataset. This data set contains four distinct types of product reviews extracted from Amazon.com, including Books (B), DVDs (D), Electronics (E), and Kitchen Appliances (K). Each review comes with full text and the rating score from the reviewer. As in [29], 800 reviews have been selected randomly from Amazon product reviews dataset, 200 of each domain. Then, we employ Google Translate (GT) to translate the test data to the target language and manually correct the output. Table 1 summarizes the dataset characteristics.

To measure the performance of sentiment classification methods, experimental results are presented using the gold standard statistical metrics used in machine learning that include: True Positive (TP) of a class is the set of reviews that are correctly assigned to that class, False Positive (FP) of a class is the set of reviews that are incorrectly assigned to that class, False Negative (FN) of a class is the set of reviews that is incorrectly rejected for corresponding class, and True Negative (TN) is the set of reviews that is correctly rejected to that class. Precision, recall, and F1 are used to measure performances.

## 5. Result and Discussion

The following experiments are conducted using the aforementioned dataset and validation metrics (1) baseline ensemble models where experiments are conducted to evaluate the baseline ensemble model (2) baseline semi-supervised learning, and (3) the proposed integrated model. All the experiments outlined are with consistent model configurations, training and test data.

**Figure 2:**
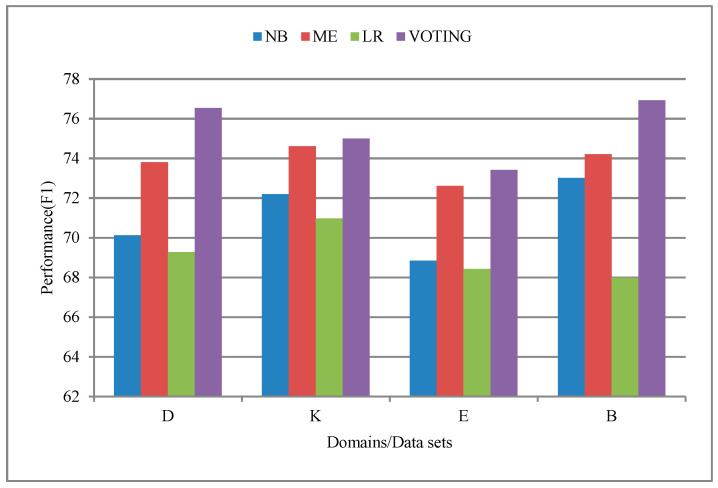
Performance of baseline supervised learning models on Books B, DVDs D, Electronics E, and Kitchen K domains.

The first set of experiments is to evaluate Supervised Cross-Lingual Learning sentiment analysis (SCLL). This means that these experiments use only translated data for the training process. Initially, LR, NB, and ME and voting ensemble classifiers are trained using translated data from English and Arabic dataset. The experimental results using LR, NB, ME, classifiers voting ensemble on Books B, DVDs D, Electronics E, and Kitchen Appliances K are summarized in Table 2 and Figure 2. Table 2 indicates that the highest performance is obtained with the voting ensemble classifier with f-measure performance of 76.54%, 75%, 73.42%, and 76.92% on Books B, DVDs D, Electronics E, and Kitchen Appliances K, respectively. On the other hand, the LR classifiers show poor classification accuracy with f-measure performance of 69.28%, 70.97%, 68.42%, and 67.98% on Books B, DVDs D, Electronics E, and Kitchen Appliances K, respectively. The outcomes from ensemble model clearly indicate the superiority over the performances of individual classifiers. This further indicates the independence between the predictions from individual classifiers.

Figure 2 demonstrates that the performance of the baseline classifiers varies from domain to domain. However, generally, the results obtained by the proposed model are significantly better than those obtained by supervised in-lingual adaptation. We argue that this is because different term distribution between original and translated documents can lead to low performance in cross-lingual sentiment classification.

The second experiment is to evaluate the Semi-Supervised Learning (SSL) sentiment analysis that only uses seeds from the target language. The co-training semi-supervised learning method is evaluated. Results are also provided in Table 3. Table 3 shows that the highest performance is obtained with the SSL with f-measure performance of 62.07%, 64.41%, 66.67%, and 63.01% on Books B, DVDs D, Electronics E, and Kitchen Appliances K respectively. From Table 2 and Table 3, the results show that SCLL trained with large translated data from the source language is superior to the SSL with seed from the target language.

In addition to the evaluation of the baseline models, the paper aims to answer how SCLL models trained with the selection of training sample from translated sentiment sources to be employed together with the target data by SSL to successfully solve cross-language analysis tasks. To do this, we investigate the effect and importance of different sizes of selected samples for cross-lingual sentiment classification. Furthermore, experiments also investigate the integrated model to show the importance of the exploitation of monolingual resources for cross-lingual sentiment classification.

**Figure 3:**
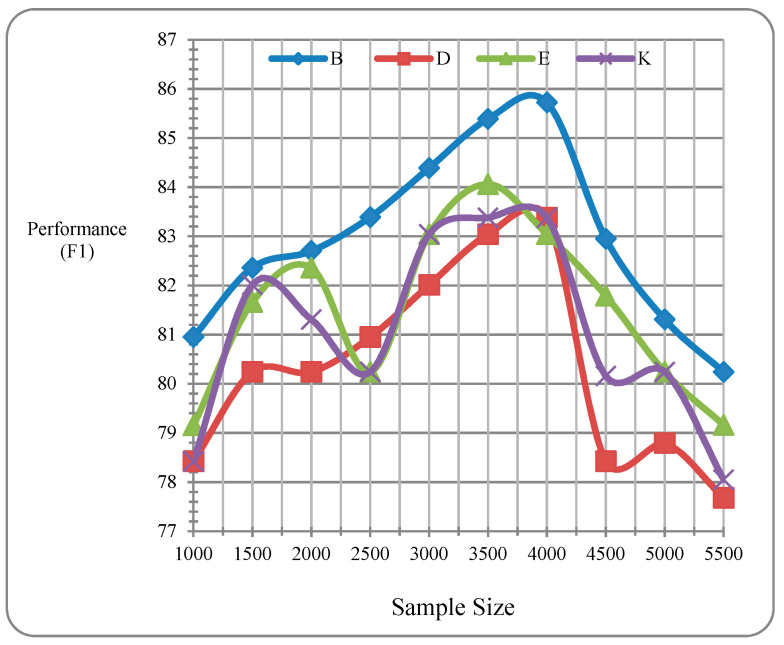
Performance (*x*-axis) of integrated supervised and semi-supervised learning models with different sizes of samples (*y*-axis).

Table 4 shows the overall performance of the integrated model proposed in this study. Results clearly indicate that the integrated learning model that combines SCLL and SSL and utilizes monolingual resources substantially improves the overall performance over baseline models. Figure 3 show the performance (*x*-axis) of an integrated supervised and semi-supervised learning model with different sizes of samples (*y*-axis).

Table 4 show that the highest performance is obtained with the integrated model when sample size is 4000 with f-measure performances of 85.72%, 83.38%, 83.04%, and 85.72% on Books B, DVDs D, Electronics E, and Kitchen Appliances K respectively. These results are significantly better than that of the best baseline models (voting ensemble classifier) with f-measure performances of 76.54%, 75%, 73.42%, and 76.92% on Books B, DVDs D, Electronics E, and Kitchen Appliances K respectively.

Based on the statistical results shown in Table 2, Table 3 and Table 4, it can be validated that the optimal selection of resources and the appropriate integration of SCLL and SSL significantly improve the performance of cross-lingual sentiment analysis.

## 6. Conclusions

A study on cross-lingual sentiment analysis using integrated supervised and semi-supervised models is presented in this paper. The aim is to show that SCLL models trained with selected training sample from translated sentiment sources can be integrated together with the target data by SSL to successfully solve cross-language analysis tasks. We designed and developed a clustering-based sample selection approach and a target-based feature selection to select the optimal and representative training samples and features that are suitable for the target data. Several experiments are conducted to evaluate standalone supervised or semi-supervised Cross-Lingual Learning sentiment analysis as well as the proposed model. Results show that SCLL trained with large translated data from the source language is superior to the SSL with seed from the target language. Experimental results also indicated that the proposed integrated models (supervised and semi-supervised models) are much more accurate than standalone supervised or semi-supervised machine learning approaches. In addition, our work showed that the majority voting method has a stable performance in the presence of noise. This paper concludes that the appropriate selection of resources and the integration of SCLL and SSL can handle cross-lingual sentiment analysis problems.

Future work will involve the use of other language pairs as well as investigating other semi-supervised learning models.

## Figures and Tables

**Table 1:** Characteristics of The Data Set.

Dataset/Features	Books	DVDS	Electronics	Kitchen
No. of reviews	2000	2000	2000	2000
Positive	1000	1000	1000	1000
Negative	1000	1000	1000	1000
No of features	188,050	179,879	104,027	89,478
Average length/review	239	234	153	131

**Table 2:** Performance of baseline supervised learning models.

Model	Target Data Set	Precision	Recall	F-Measure
NB	D	70.59	69.68	70.13
	K	72.44	71.97	72.2
	E	68.18	69.54	68.85
	B	74.19	71.88	73.02
ME	D	74.05	73.58	73.81
	K	74.84	74.38	74.61
	E	73.08	72.15	72.61
	B	73.75	74.68	74.21
LR	D	67.95	70.67	69.28
	K	70.06	71.9	70.97
	E	69.33	67.53	68.42
	B	67.76	68.21	67.98
VOTING	D	77.5	75.61	76.54
	K	76.43	73.62	75
	E	74.36	72.5	73.42
	B	77.16	76.69	76.92

**Table 3:** Performance of Baseline Semi-Supervised Learning Model.

Target Data Set	Precision	Recall	F-Measure
D	65.07	63.76	64.41
K	63.89	62.16	63.01
E	67.11	66.23	66.67
B	63.38	60.81	62.07

**Table 4:** Results (F-Measure) of Integrated Model with different sizes of selected samples.

Size of Sample	B	D	E	K
1000	80.95	78.42	79.15	80.95
1500	82.36	80.24	81.66	82.36
2000	82.7	80.24	82.36	82.7
2500	83.39	80.95	80.24	83.39
3000	84.39	82.01	83.04	84.39
3500	85.39	83.04	84.06	85.39
4000	85.72	83.38	83.04	85.72
4500	82.95	78.42	81.79	80.16
5000	81.31	78.79	80.24	80.24
5500	80.24	77.68	79.16	78.05
